# Significant Geometry Features in Tongue Image Analysis

**DOI:** 10.1155/2015/897580

**Published:** 2015-07-13

**Authors:** Bob Zhang, Han Zhang

**Affiliations:** Department of Computer and Information Science, University of Macau, Taipa, Macau

## Abstract

The shape of a human tongue and its relation to a patients' state, either healthy or diseased (and if diseased which disease), is quantitatively analyzed using geometry features by means of computerized methods in this paper. Thirteen geometry features based on measurements, distances, areas, and their ratios are extracted from tongue images captured by a specially designed device with color correction. Using the features, 5 tongue shapes (rectangle, acute and obtuse triangles, square, and circle) are defined based on traditional Chinese medicine (TCM). Classification of the shapes is subsequently carried out with a decision tree. A large dataset consisting of 672 images comprising of 130 healthy and 542 disease examples (labeled according to Western medical practices) are tested. Experimental results show that the extracted geometry features are effective at tongue shape classification (coarse level). Even if more than one disease class belongs to the same shape, the disease classes can still be discriminated via fine level classification using a combination of the geometry features, with an average accuracy of 76.24% for all shapes.

## 1. Introduction

The human tongue contains numerous features. Traditionally, medical practitioners would examine these features based on years of experience [1–5]. However, ambiguity and subjectivity are associated with their diagnostic results. To eliminate these qualitative aspects, tongue images can be objectively analyzed, which offers a new way to diagnose disease, one that minimizes the physical harm inflicted to patients (compared with other medical examinations).

In state-of-the-art computerized tongue image analysis, color and texture features are the most prevalent [6–19]. There exists little or no literature on tongue image analysis using geometry features, whereas in traditional medicines such as traditional Chinese medicine (TCM) the shape of a tongue can be used to determine a patients' illness [3, 4, 20]. The authors in [21] proposed an approach to automatically recognize tongue shapes based on geometry features. The seven geometric features included various measurements of length, area, and angle extracted from tongue images. Using a support decision tool to weight the relative influences of the geometry features, they classified an image into one of six tongue shapes, namely, hammer, rectangle, acute triangle, obtuse triangle, square, and round (based on TCM). Experimental results conducted on 362 tongue images exhibited an accuracy of 90.3% for shape classification. Nevertheless, there was little quantitative analysis between tongue shape and the relationship to its current health state.

**Figure 1:**
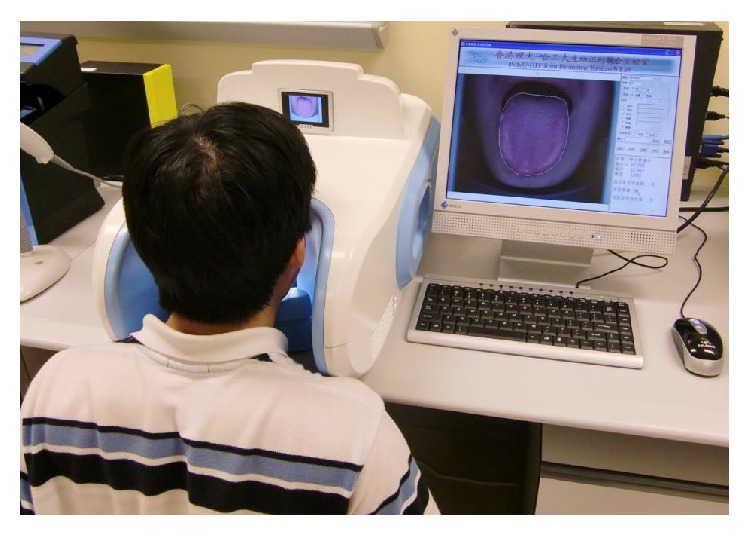
Tongue image capture device.

In this paper we thoroughly examine the aforementioned problem via geometry features in tongue image analysis. The imaging device used to capture tongue images is made up of a 3-chip CCD camera with 8 bit resolution and two D65 fluorescent tubes placed symmetrically around the camera in order to produce a uniform illumination. The images captured were color corrected [22] to eliminate any noise caused by variations of illumination and device dependency. Also, the tongue image capture device ensures that the images are properly aligned. This allows consistent feature extraction and classification in the following steps.  Figure 1 shows the capture device. Using this device we form a large tongue image database consisting of 672 samples. This database is composed of 130 healthy and 542 disease samples, divided into 7 classes with at least 19 examples. Every image is segmented [19] with the background removed and tongue foreground remaining. From each tongue image consisting of a tip, body, and root [20], 13 geometry features derived from measurements, distances, areas, and their ratios are extracted. Using these features we define 5 tongue shapes based on TCM, rectangle, acute and obtuse triangles, square, and circle. Coarse level classification applying a decision tree [23] was used to classify a tongue into one of the shapes. Experimental results showed that a majority of the samples in the classes from healthy and disease samples tend to be one form, where healthy versus disease samples and disease versus disease are separable using fine level classification, employing a combination of geometry features. This proves the significance of the geometry features at establishing a relationship between a tongue's state and its shape.

The rest of this paper is organized as follows. Thirteen geometry features extracted from tongue images are presented in Section 2 along with the tongue shape classification performed using a decision tree. Following this, experimental results using coarse and fine level classifications are given in Section 3. Finally, concluding remarks are made in Section 4.

## 2. Materials and Methods

The tongue image dataset is first introduced in Section 2.1. Afterwards, a detailed description of the 13 geometry features extracted from a tongue image is given in Section 2.2. How these features are then used to classify a tongue into 5 major shapes is provided in Section 2.3.

### 2.1. Tongue Image Dataset

The tongue image database is composed of 672 images (one image per person) divided into 130 healthy and 542 disease samples. Seven disease classes and healthy classes were captured at Guangdong Provincial Hospital of Traditional Chinese Medicine, Guangdong, China. Patients with diabetes mellitus were processed at the Hong Kong Foundation for Research and Development in Diabetes, Prince of Wales Hospital, Hong Kong. Healthy samples were verified through a blood test and other experiments. If indicators from the tests fall within a certain range they were deemed healthy. In the disease class, samples were collected from inpatients with illness determined by their admission note and diagnosed using western medical practices. Inpatients suffering from the same disease were grouped together into a single class. In total there were 7 disease groups (with at least 19 samples). A summary of the disease class breakdown is given in Table 1.

### 2.2. Geometry Features

In the following subsection we describe the 13 geometry features extracted from tongue images (which have been converted to binary images after segmentation [19]). These features based on measurements, distances, areas, and their ratios are used in subsequent sections to define and classify 5 tongue shapes.

#### 2.2.1. Width

**Figure 2:**
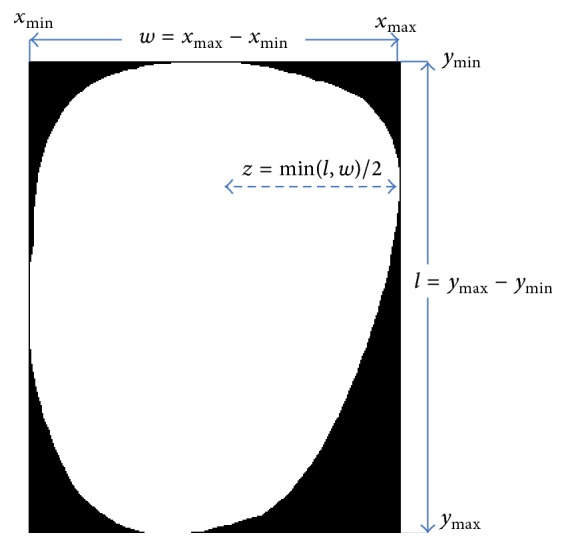
Illustration of features 1, 2, and 4.

The width (*w*) feature (see Figure 2) is measured as the horizontal distance along the *x*-axis from a tongue's furthest right edge point (*x*_max_) to its furthest left edge point (*x*_min_):

(1) $$\begin{array}{c} w=x_{max}-x_{min}. \end{array}$$

#### 2.2.2. Length

The length (*l*) feature (see Figure 2) is measured as the vertical distance along the *y*-axis from a tongue's furthest bottom edge (*y*_max_) point to its furthest top edge point (*y*_min_):

(2) $$\begin{array}{c} l=y_{max}-y_{min}. \end{array}$$

#### 2.2.3. Length-Width Ratio

The length-width ratio (*lw*) is the ratio of a tongue's length to its width:

(3) $$\begin{array}{c} lw=\frac{l}{w}. \end{array}$$

#### 2.2.4. Smaller Half Distance

Smaller half distance (*z*) is the half distance of *l* or *w* depending on which segment is shorter (see Figure 2):

(4) $$\begin{array}{c} z=\frac{min\left(l,w\right)}{2}. \end{array}$$

#### 2.2.5. Center Distance

**Figure 3:**
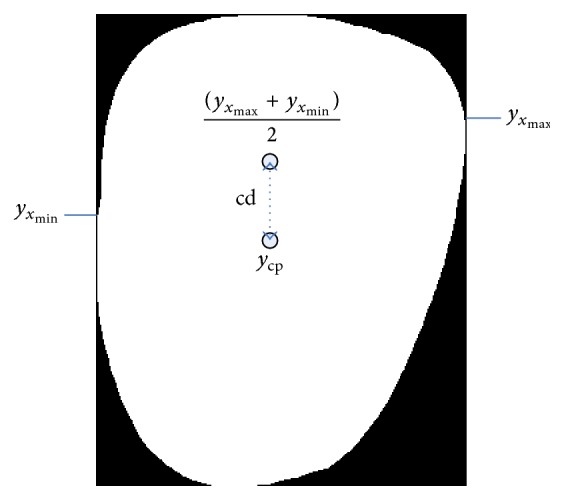
Illustration of feature 5.

The center distance (cd) (refer to Figure 3) is the distance from *w*'s *y*-axis center point to the center point of *l*(*y*_cp_):

(5) $$\begin{array}{c} cd=\frac{\left(max\left(y_{x_{max}}\right)+max\left(y_{x_{min}}\right)\right)}{2}-y_{cp}, \end{array}$$

where *y*_cp_ = (*y*_max_ + *y*_min_)/2.

#### 2.2.6. Center Distance Ratio

Center distance ratio (cdr) is ratio of cd to *l*:

(6) $$\begin{array}{c} cdr=\frac{cd}{l}. \end{array}$$

#### 2.2.7. Area

The area (*a*) of a tongue is defined as the number of tongue foreground pixels.

#### 2.2.8. Circle Area

**Figure 4:**
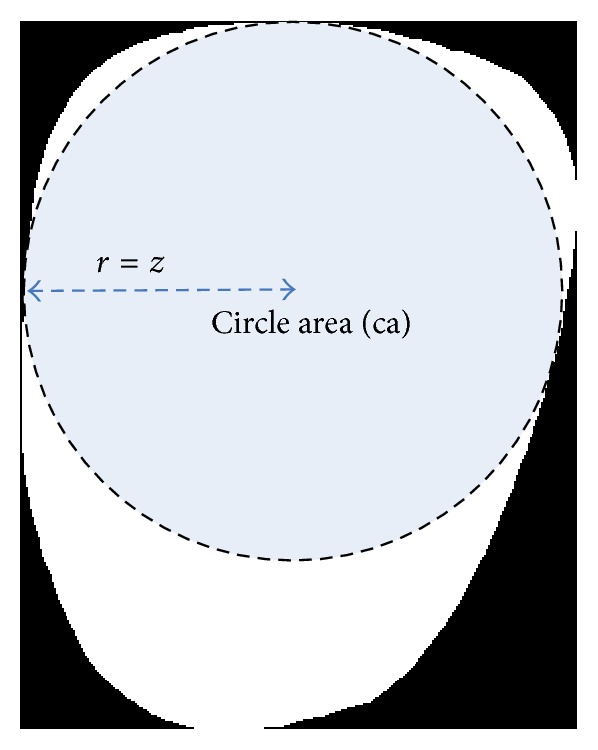
Illustration of feature 8.

Circle area (ca) is the area of a circle within the tongue foreground using smaller half distance *z*, where *r* = *z* (refer to Figure 4):

(7) $$\begin{array}{c} ca=\pi r^{2}. \end{array}$$

#### 2.2.9. Circle Area Ratio

Circle area ratio (car) is the ratio of ca to *a*:

(8) $$\begin{array}{c} car=\frac{ca}{a}. \end{array}$$

#### 2.2.10. Square Area

**Figure 5:**
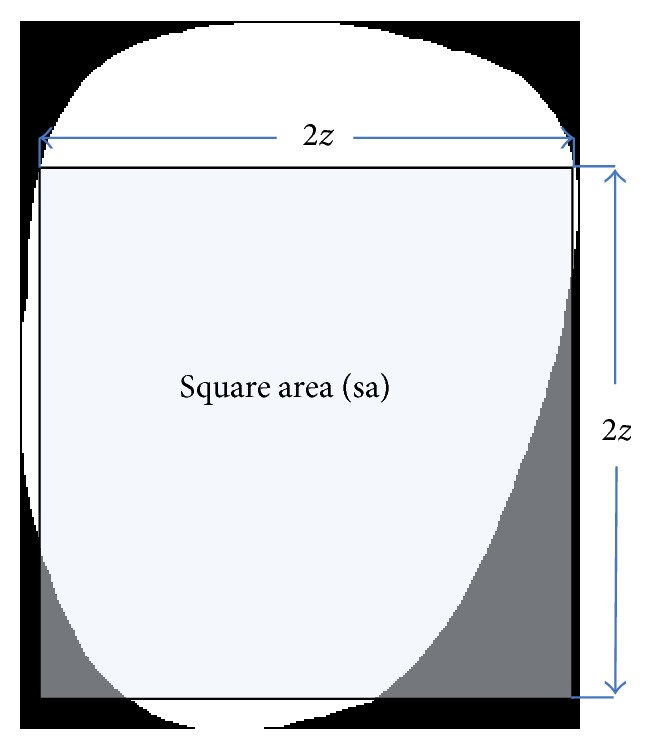
Illustration of feature 10.

Square area (sa) is the area of a square defined within the tongue foreground using smaller half distance *z* (refer to Figure 5):

(9) $$\begin{array}{c} sa=4z^{2}. \end{array}$$

#### 2.2.11. Square Area Ratio

Square area ratio (sar) is the ratio of sa to *a*:

(10) $$\begin{array}{c} sar=\frac{sa}{a}. \end{array}$$

#### 2.2.12. Triangle Area

**Figure 6:**
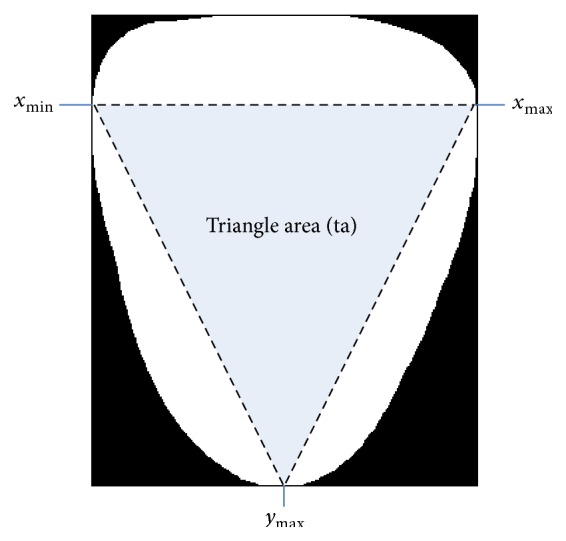
Illustration of feature 12.

Triangle area (ta) is the area of a triangle defined within the tongue foreground (see Figure 6). The right point of the triangle is *x*_max_, the left point is *x*_min_, and the bottom is *y*_max_.

#### 2.2.13. Triangle Area Ratio

Triangle area ratio (tar) is the ratio of ta to *a*:

(11) $$\begin{array}{c} tar=\frac{ta}{a}. \end{array}$$

### 2.3. Tongue Shape Classification

**Figure 7:**
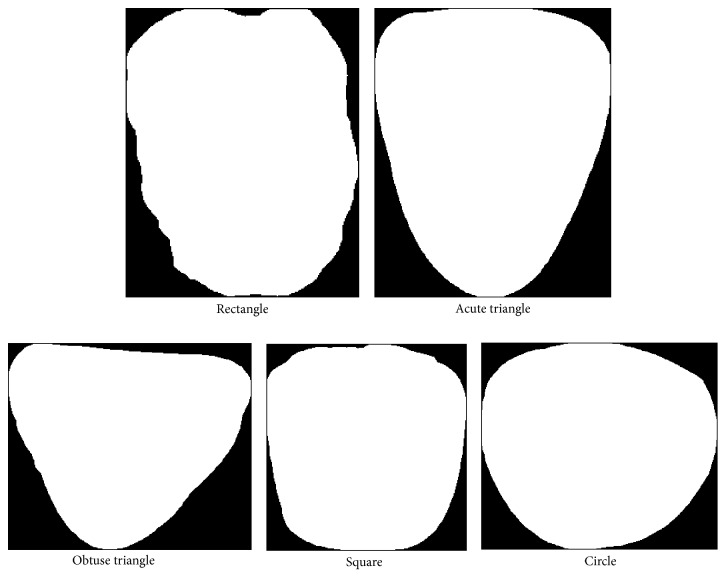
Typical samples to show the 5 tongue shapes.

Based on TCM we define 5 tongue shapes, rectangle, acute triangle, obtuse triangle, square, and circle, which can be classified using the 13 features explained above (see Section 2.2). A rectangle tongue's vertical length is long, but its horizontal width along the tip, body, and root remains relatively constant. An acute triangle tongue's vertical length is longer than its largest horizontal width (at the root) but gradually decreases from the body down to the tip. If the tongue shape is an obtuse triangle, its horizontal width is greater than its vertical length, with the width steadily decreasing as it approaches the tip. In a square tongue shape both its horizontal width and vertical length are similar. Finally, if a tongue is circle, both the horizontal width and vertical length will be alike, but its car (8) will be closer to 1.  Figure 7 depicts the 5 tongue shapes using typical samples.

**Figure 8:**
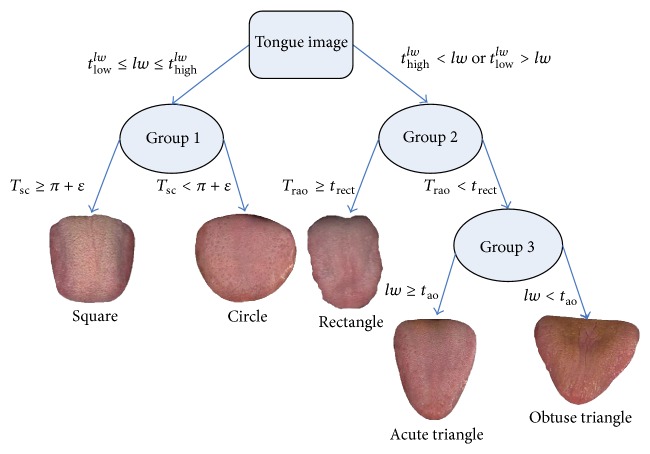
Decision tree to classify the tongue shapes.

To classify tongue images into its proper shape, a decision tree structure shown in Figure 8 is used. Given a tongue we first examine its length-width (*lw*) ratio. If this ratio is *t*_low_^*lw*^ ≤ *lw* ≤ *t*_high_^*lw*^, the tongue shape must be square or circle (left branch), and if the ratio is *t*_high_^*lw*^ < *lw* or *t*_low_^*lw*^ > *lw*, the shape of the tongue can be rectangle, acute triangle, or obtuse triangle (right branch). The values of *t*_low_^*lw*^ and *t*_high_^*lw*^ are 0.95 and 1.05, respectively.

Focusing on the left branch, the average radius (*r*_avg_) of the tongue is first calculated as

(12) $$\begin{array}{c} r_{avg}=\frac{\left(l+w\right)}{4} \end{array}$$

which is the average of *w*/2 and *l*/2. Next, the ratio *T*_sc_ is computed as

(13) $$\begin{array}{c} T_{sc}=\frac{a}{{r_{avg}}^{2}}. \end{array}$$

If the tongue shape is approximately square, the value of *T*_sc_ ≈ 4  (i.e., 4 · *r*_avg_^2^/*r*_avg_^2^), and if it is approximately circle, *T*_sc_ ≈ *π* (i.e., *π*·*r*_avg_^2^/*r*_avg_^2^). Hence, the two shapes can be defined as

(14) $$\begin{array}{c} Square=T_{sc}\geq \pi +\varepsilon ,\\ Circle=T_{sc}<\pi +\varepsilon , \end{array}$$

where *ε* is a constant equal to 0.1.

Turning our attention to the right branch, we initially calculate the ratio *T*_rao_:

(15) $$\begin{array}{c} T_{rao}=\frac{a}{\left(l\cdot w\right)}. \end{array}$$

If this ratio is greater than or equal to *t*_rect_, the tongue shape is rectangle:

(16) $$\begin{array}{c} Rectangle=T_{rao}\geq t_{rect}, \end{array}$$

where *t*_rect_ is 0.85 and the maximum of *T*_rao_ is 1. If *T*_rao_ < *t*_rect_, the shape of the tongue is either acute or obtuse triangle. To determine which triangle, length-width ratio is used once again as follows:

(17) $$\begin{array}{c} Acute\;Triangle=\left(T_{rao}<t_{rect}\right)\wedge \left(lw\geq t_{ao}\right),\\ Obtuse\;Triangle=\left(T_{rao}<t_{rect}\right)\wedge \left(lw<t_{ao}\right), \end{array}$$

where *t*_ao_ is given as 1.05. The parameter values listed above to classify a tongue image were chosen empirically.

## 3. Results and Discussions

The following section presents the experimental results. A coarse level classification showing the results of tongue shape classification is given in Section 3.1. Classes classified into the same shape are further differentiated in Section 3.2 through fine level classification using a combination of geometry features.

### 3.1. Coarse Level: Tongue Shape Classification Result

**Figure 9:**
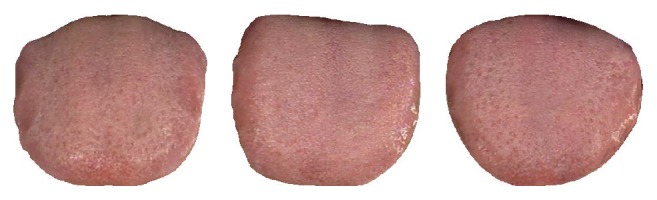
Three typical samples from healthy samples.

**Figure 10:**
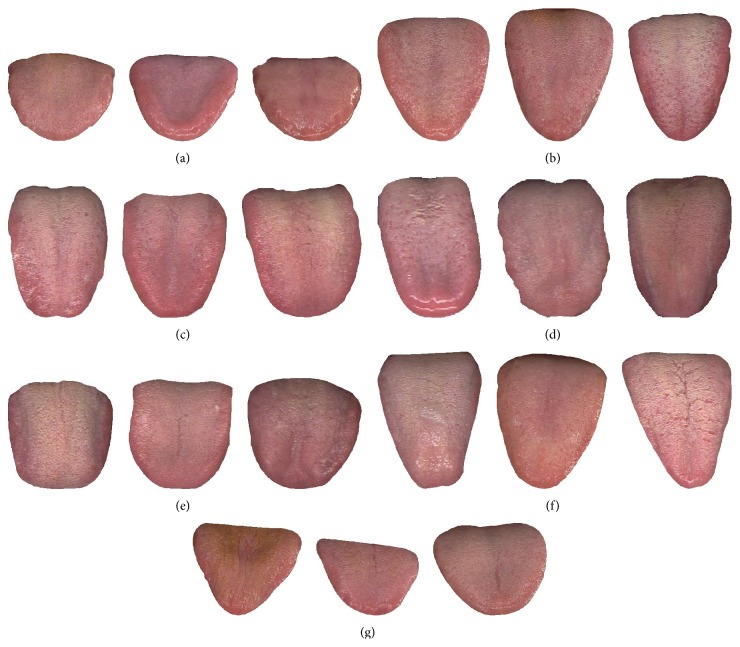
Three typical samples from (a) DM, (b) NR, (c) GV, (d) NS, (e) EG, (f) CG, and (g) CHD.

By applying the tongue shape classification algorithm (described above, see Section 2.3) to every image in the dataset, its shape can be determined. This result is listed in Table 2. In the table it can be seen that the most common shapes in healthy group are circle or square, representing 86.92% (113/130) of all samples. In DM the majority shape is obtuse triangle, accounting for 86.15% (255/296). For NR, acute triangle takes the majority with 83.33% (75/90). Having 85.07% (57/67) and 83.33% (25/30), rectangle is the most prevalent shape in GV and NS, respectively. In EG the dominant shapes are circle or square, making up 85.00% (17/20). Finally, in CG and CHD acute and obtuse triangles are the most widespread, embodying 80.00% (16/20) and 84.21% (16/19) of all images correspondingly.  Figure 9 depicts three typical samples from the healthy class, while Figures 10(a)–10(g) illustrate typical samples from the disease classes.

### 3.2. Fine Level: Classification Result within Each Shape

In the tongue shape classification results, there exists more than one class for each shape (see Table 2). To distinguish between classes with the same shape, a set of geometry features were selected and applied to SVM. Half the images in each class were randomly selected for training, while the other half was used as testing. To measure the performance, average accuracy was employed. The linear kernel function (dot product) was used to map the training data into kernel space, while a quadratic kernel produced similar classification results. *k*-NN was also tested but did not perform as well.

Utilizing a grouping of the features is logical, since not every feature can have a positive contribution to the final result, as is the case here where 13 features used for classification produced poor results. Therefore, an optimization of the features is necessary. To reduce the number of features, sequential forward selection (SFS) [23] was implemented. SFS is a feature selection method that begins with an empty set of features. It adds additional features based on maximizing some criterion function *J* and terminates when all features have been added. In our case *J* is the average accuracy of SVM. Below, each tongue shape is examined in detail.

#### 3.2.1. Rectangle

Both GV and NS were classified into this shape. Applying SFS with SVM to separate the two classes, the highest average accuracy of 70.07% was achieved using features 9, 4, 7, 8, 2, 13, 12, and 1.

#### 3.2.2. Acute Triangle

For this shape NR and CG were classified together. *l* (feature 2) attained the best average accuracy of 70.00%.

#### 3.2.3. Obtuse Triangle

DM and CHD were assigned to obtuse triangle. Through a combination of features consisting of 1, 7, 4, 11, 13, and 2, the highest average accuracy of 76.23% was achieved.

#### 3.2.4. Circle or Square

The maximum average accuracy of 88.65% was obtained using *w* (feature 1) and *a* (feature 7) to classify the two classes (healthy and EG) appointed to circle or square.

Table 3 summarizes the results for each tongue shape. For completeness, the average accuracy of healthy samples versus NR, GV, NS, CG, DM, and CHD is shown in Table 4. From this result, it can be seen that healthy samples are distinguishable compared to others, given their different tongue shapes.

## 4. Conclusions

In this paper we thoroughly examined tongue shape and its relation to a patients' state (either healthy or diseased) using geometry features through computerized methods. With tongue images captured by a specially designed device that accounts for image correction and a large dataset labeled according to western medical practices, we have a solid foundation to carry out this objective study. Thirteen geometry features including measurements, distances, areas, and their ratios were extracted from each tongue image. The features helped define 5 tongue shapes rooted upon TCM and classified using a decision tree. In the experimental results, coarse level classification first showed that the tongue classes belong to different shapes. Although more than one class occupies the same shape, in fine level classification they are still distinguishable, when employing SFS with SVM (using a grouping of geometry features). This validates the significance of geometry features at shape classification, as well as healthy versus disease/disease versus disease classifications. With tongue shape and a persons' health state now established using computer-based methods, this potentially provides a new painless and efficient way to diagnose patients. A continuation of this work will investigate the fusion of all possible tongue features including color and texture in order to better determine a patients' state.

## Figures and Tables

**Table 1:** Disease class statistics listing its name and number of samples.

Disease name	Number of samples
Diabetes mellitus (DM)	296
Nephritis (NR)	90
Gastritis verrucosa (GV)	67
Nephrotic syndrome (NS)	30
Erosive gastritis (EG)	20
Chronic gastritis (CG)	20
Coronary heart disease (CHD)	19

**Table 2:** Tongue shape classification result for the dataset.

	Rectangular	Acute triangle	Obtuse triangle	Circle	Square
Healthy	5	4	8	**50**	**63**
DM	7	22	**255**	2	10
NR	5	**75**	4	0	6
GV	**57**	5	4	0	1
NS	**25**	1	1	1	2
EG	1	1	1	**5**	**12**
CG	4	**16**	0	0	0
CHD	0	3	**16**	0	0

**Table 3:** Optimized classification result between various classes having identical tongue shape.

Tongue shape	Class comparison	Optimal feature(s)	Average accuracy
Circle or square	Healthy versus EG	1, 7	88.65%
Obtuse triangle	DM versus. CHD	1, 7, 4, 11, 13, 2	76.23%
Acute triangle	NR versus CG	2	70.00%
Rectangle	GV versus NS	9, 4, 7, 8, 2, 13, 12, 1	70.07%

**Table 4:** Optimized classification result between healthy versus NR, GV, NS, CG, DM, and CHD groups.

Classes compared with healthy	Optimal feature(s)	Average accuracy
NR	1, 7, 12, 8, 4	82.09%
GV	1, 8	86.72%
NS	7, 4, 1, 8	85.26%
CG	1	75.19%
DM	12, 1, 13, 9, 11	69.09%
CHD	8, 6, 4, 10, 13, 9, 2	80.89%
